# Reconfigurable Microwave Absorption Properties and Principles of Double-Layer Metasurface Absorbers

**DOI:** 10.3390/molecules30173608

**Published:** 2025-09-03

**Authors:** Yun He, Zhiming Zhang, Qingyang Wang, Qiyuan Wang, Qin Fu, Yulu Zhang

**Affiliations:** 1 School of Information Engineering, Wuhan University of Technology, Wuhan 430070, China; heyun@whut.edu.cn (Y.H.); zhangzhiming@whut.edu.cn (Z.Z.); 18973418795@163.com (Q.W.); w2729130943@163.com (Q.W.); 2 School of Integrated Circuits, Nanjing University of Information Science and Technology, Nanjing 210044, China

**Keywords:** microwave absorber, wideband, reconfigurable, metasurface

## Abstract

A reconfigurable microwave absorber based on double-layer metasurface is proposed for wide microwave band applications spanning 3 to 14 GHz. The absorber consists of two layers with two-dimensional array of four-semi-circular and square-ring metasurface patches loaded impedance devices, two spacers composed of honeycomb materials, and a bottom copper substrate. In order to break through the limitation of single-layer absorbers at finite resonant frequencies, a special double-layered metasurface structure is adopted. The layer I of metasurface is designed with two resonant peaks near the X band and transmission performance in the C band. Simultaneously, the layer II of metasurface is designed with a resonant peak near the C band and reflection performance in the X band. To achieve a reconfigurable effect, impedance adjustable device, such as PIN diodes, are connected between patterned metasurface cells of layer I. The simulation results revealed that the double-layer metasurface absorber can not only achieve broadband absorption effect, with the reflection value below −10 dB from 3.1 to 14.2 GHz, but also adjust the electromagnetic absorption rate, with the reflection value below −20 dB covers a bandwidth of 6.6–9 GHz. The good agreement between simulation and measurement validates the proposed absorber.

## 1. Introduction

Microwave absorbers such as frequency selective surfaces [1,2], metamaterials [3,4,5], and metasurfaces [6,7,8,9], have been gaining intense interest over recent years. Especially, metasurfaces are increasingly widely used in the field of electromagnetic regulation due to their flexibility in regulating the amplitude [10], phase [11], polarization [12], and other characteristics of electromagnetic waves [13,14]. Recently, with the advancement of metamaterial theory, metasurfaces have demonstrated broad application prospects in stealth technology [15,16], electromagnetic compatibility [17], and mobile communications due to their structural flexibility, lightweight low-profile design, and customizable absorption properties [18]. Current research on metasurface absorbers primarily focuses on broadband performance [19], multi-band compatibility [20], and polarization-insensitive designs [21]. For example, Han et al. proposed a metasurface absorber consisting of patterned graphene sandwich structure, transparent flexible polyvinyl chloride dielectric layer, and indium tin oxide bottom plate; the RCS reduction in the conformal case is better than 10 dB in 8.52–16.98 GHz, and the corresponding relative bandwidth is 66.35% [22]. Gong et al. proposed a chessboard-shaped polarization conversion metasurface to significantly reduce the radar cross-section [23]. Therefore, enhancing the broadband performance of the microwave absorber is of great significance.

To realize the broadband performance of metasurface absorbers, the design is mainly achieved through two approaches: multilayer structures and tunable devices [24,25,26,27,28]. Zou et al. proposed a 12 mm multilayer dielectric gradient honeycomb absorber with a double layer metasurface consisting of a resistive film and an artificial magnetic conductor checkerboard [26]. Basiri et al. designed a three-layer absorber including a graphene metasurface sheet, Topas-cyclic olefin copolymer dielectric substrate, and a gold ground plane [28]. Multilayer absorbers expand the operational bandwidth and enhance absorption efficiency by leveraging the synergistic effects of multiple resonances and loss mechanisms across different layers, such as impedance-matching layers and lossy layers [29,30,31,32,33]. Cao et al. demonstrated a wide-angle high absorption solar selective absorber based on the impedance matching between the absorber and the free space, which can maintain the average omnidirectional absorption over the incident angle ranges from 8 to 80° [33]. However, multilayer designs face challenges in precisely controlling interlayer coupling effects. Additionally, the inherent resonant frequency limitations of passive metasurface structures are difficult to overcome with multilayer configurations alone.

Tunable absorbers incorporate dynamic modulation mechanisms, such as electrical [34], optical [35], or mechanical tuning [36], to reconfigure absorption properties and meet broadband absorption demands in complex electromagnetic environments [37,38,39]. Typical methods include integrating tunable impedance components such as PIN diodes, functional materials such as liquid crystals [40,41], or deformable structures such as MEMS [42,43]. These absorbers excel in frequency agility, polarization switching, and absorption intensity modulation. However, tunable designs in multilayer structures may disrupt the original interlayer coupling effects, thereby influencing the tuning efficacy of absorption performance.

In this paper, we propose and experimentally validate a reconfigurable double-layer metasurface absorber targeting wideband microwave absorption from 3 to 14 GHz. To circumvent the inherent bandwidth constraints of single-layer absorbers reliant on discrete resonances, our design leverages a configured dual-metasurface architecture. This architecture comprises two distinct metasurface layers (layer I and layer II). Crucially, layer I is engineered to provide resonant absorption in the X band while exhibiting transmission properties in the C band. Conversely, layer II is designed for resonant absorption in the C band while reflecting incident waves in the X band. This complementary resonant mechanism facilitates synergistic broadband absorption across the target spectrum. Furthermore, to achieve dynamic reconfigurability addressing the limitations of purely passive multilayer designs, impedance-tunable PIN diodes are integrated into the patterned metasurface unit cells of layer I. A comprehensive analysis of the induced surface current distribution of the typical resonant absorption peaks provides a basis to understand the microwave absorption mechanism of the metasurface absorber in detail. Both the simulation and measurement results have verified the wideband and tunable absorption performance of this design scheme.

## 2. Results and Discussion

Following the structural design, the electromagnetic performance of the proposed absorber was investigated through rigorous full-wave simulations using the High Frequency Structure Simulator (HFSS). The bottom layer of the designed structure is a metal plane and the transmitted electromagnetic energy is very small. Therefore, we usually can use the dB value of the reflectivity to characterize the absorption performance of the structure. This section presents the key findings from these HFSS simulations and the corresponding experimental validation measurements. The results characterize the absorber’s absorption performance, providing a foundation for the subsequent analysis and discussion in the following sections.

### 2.1. Design of the Integrated Structure

**Figure 1:**
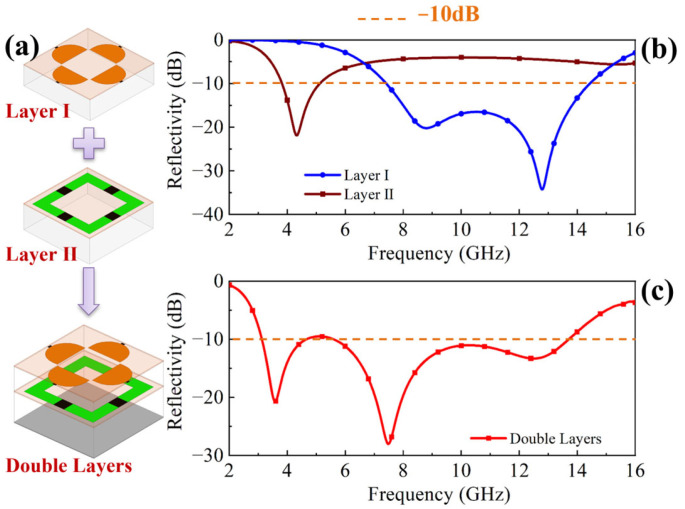
Design procedure of the double-layer structure. (**a**) Integration of the two functional layers; simulation results of reflectivity curves of (**b**) layer I and layer II and (**c**) double-layer absorber.

Figure 1a illustrates the design methodology of the double-layer metasurface absorber, fabricated through the integration of two independent layers (layer I and layer II). The orange dotted line represents the target value −10 dB of the design. Figure 1b presents the simulated reflection coefficient for each layer, where *R_v_* is under a fixed resistance of 25 ohm. Layer I exhibits dual resonant frequencies around 8.8 GHz and 12.8 GHz, achieving broadband absorption with reflectivity below −10 dB across 7.5–14.4 GHz, while layer II demonstrates strong absorption near 4.3 GHz with a minimum reflection of −20 dB. Then the integrated dual-layer structure was simulated in HFSS. As shown in Figure 1c, the resultant reflection coefficient curve effectively combines the absorption characteristics of both individual layers, yielding three distinct resonance points and achieving reflectivity of less than −10 dB within the 3.1–13.7 GHz frequency range.

**Figure 2:**
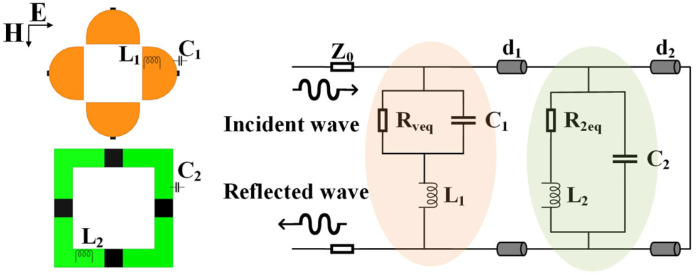
ECM of the dual-layer metasurface absorber.

An equivalent circuit model (ECM) is established to explain the design mechanism and principles in Figure 2. In the ECM on the right, the orange shaded part represents MS I while the green part indicates MS II. The *d*_1_ and *d*_2_ represent the equivalent transmission lines of the two spacer layers. Inductor *L*_1_ and *L*_2_ characterize the skin effect inductance of metallic patterns and capacitor *C*_1_ and *C*_2_ represent the intrinsic distributed capacitance between two adjacent cells. For the four symmetrically distributed resistors, their equivalent value is replaced by *R_veq_* and *R*_2*eq*_.

According to the equivalent transmission line theory, the input impedance of the transmission line that connected with loaded elements in the terminal is given by [44]:

(1) $$Z(d)=Z_{0}\frac{Z_{L}+jZ_{0}\tan(\beta d)}{Z_{0}+jZ_{L}\tan(\beta d)}$$

where *Z*_0_ is the characteristic impedance of the free space, which is equal to 377 Ω, *Z_L_* is the equivalent impedance of the loaded elements, *β* is the electromagnetic wave propagation constant, and *d* is the length of the transmission line.

The equivalent impedance of the MS I and MS II is given by:

(2) $$Z_{MS1}=Z_{R_{\mathrm{ve}q}||C_{1}}+Z_{L_{1}}=\frac{1}{\frac{1}{R_{veq}}+j\omega C_{1}}+j\omega L_{1}$$

(3) $$Z_{MS2}=Z_{(R_{2eq}+L_{2})||C_{2}}=\frac{1}{\frac{1}{R_{2eq}+j\omega L_{2}}+j\omega C_{2}}$$

Then the input impedance *Z_in_* of the dual-layer unit can be represented as [45]:

(4) $$Z_{in}=Z_{MS1}{||Z(d1)+Z}_{MS2}||Z(d2)$$

The corresponding reflection coefficient of the absorber is given by:

(5) $$\rho =\frac{Z_{in}-Z_{0}}{Z_{in}+Z_{0}}$$

Finally, the reflectivity expressed in dB values can be calculated as:

(6) Γ=20lg(ρ)

Perfect matching to free-space impedance *Z*_0_ requires *Z_in_* = *Z*_0_. The bottom layer of the designed absorber is metal, so the electromagnetic transmission rate is 0. Therefore, the electromagnetic energy incident on the absorber is distributed into two situations: absorption or reflection. The lower the reflectivity, the better the absorption performance. It can be seen from the formulas that there are many parameters for adjusting the reflectance. Considering the feasibility of parameter adjustment, variable resistors or variable capacitors are usually used to reconfigure the performance of the absorber. In this study, a variable resistor is adopted to achieve dynamic performance adjustment. The parameter of the variable resistor *R_v_* is used in the simulation and a PIN diode is used in sample preparation.

**Figure 3:**
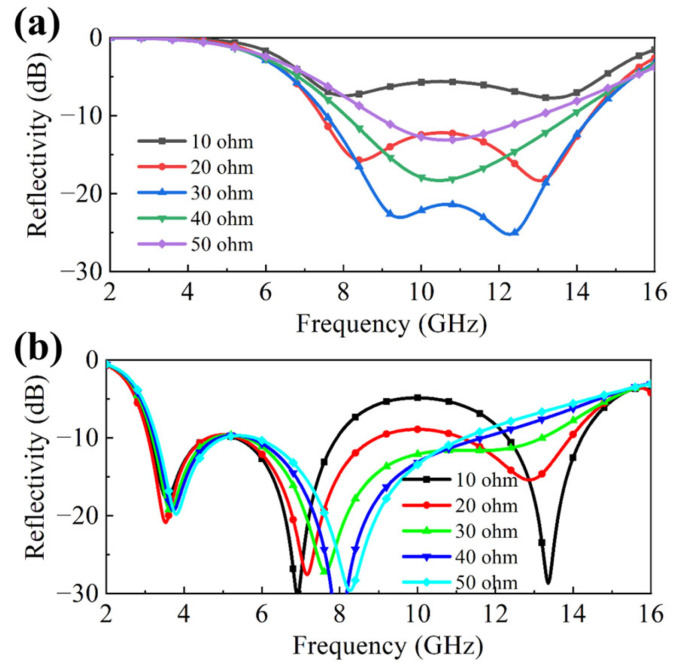
Reflectivity curves under a set of *R_v_* values. (**a**) Simulation results of single layer I absorber; (**b**) simulation results of double-layer absorber.

The preceding results preliminarily demonstrate that the absorbing performance of the double-layer absorber is achieved through the synergistic integration of the two constituent MS layers. Furthermore, the reflection coefficient can be effectively tuned by adjusting the variable resistance value (*R_v_*) within the MS layer I, implemented as a reconfigurable metasurface. To investigate this tuning mechanism, Figure 3 presents simulation results depicting the influence of varying *R_v_* on the absorber’s absorption characteristics. Specifically, Figure 3a shows the simulated reflection coefficient for single layer I absorber in Figure 1a under different *R_v_* values. As *R_v_* increases from 10 ohm to 50 ohm, a significant shift in the reflection response is observed across 7.5–14.4 GHz. Notably, both the peak value of the resonant frequency peaks and the amplitude of the reflection coefficient will change with the variation of resistance, among which the amplitude change is more obvious. Based on this, Figure 3b examines the impact of the variable resistance on the performance of the complete double-layer absorber. The double-layer metasurface absorber can achieve broadband absorption effect, with the reflection value below −10 dB from 3.1 to 14.2 GHz. It is evident that as *R_v_* continues to increase, the performance of the second resonant peak shows a significant regulating effect, maintaining a reflectivity of below −20 dB within the evaluated resistance range, continuously varying from approximately 6.6 GHz to 9 GHz. Meanwhile, the amplitude of the third resonant peak will also vary with different resistance values.

### 2.2. Analysis of the Functional Layers

**Figure 4:**
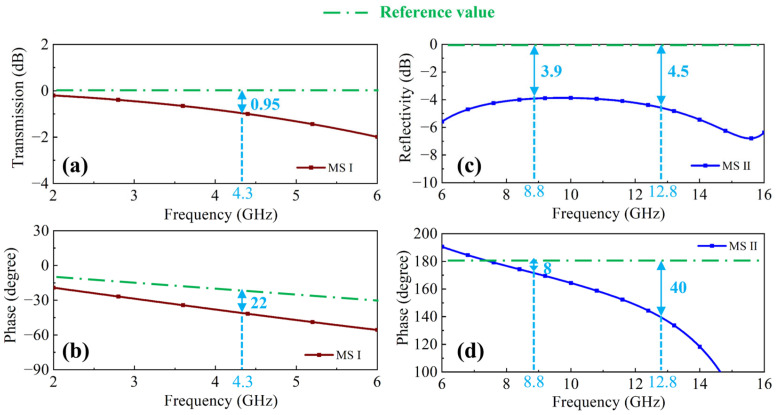
(**a**) Transmission amplitude and (**b**) transmission phase of MS I. (**c**) Reflectivity amplitude and (**d**) reflectivity phase of MS II.

It should be noted that while the reflection coefficient curve of the integrated dual-layer structure in Figure 1c captures the primary absorption characteristics of the constituent layers, slight deviations exist in both resonant frequencies and reflection coefficient magnitudes compared to the single layer performances, as shown in Figure 1b. To quantify these discrepancies, the transmission coefficient of MS I within the range of 2–6 GHz and the reflection coefficient of MS II from 6 to 16 GHz were analyzed in Figure 4.

Beyond its high-frequency absorption role, MS I is designed to function as a transmission window for the lower band (2–6 GHz). The ideal transmission reference is air, characterized by near 0 dB transmission magnitude (100% transmission) and the linear phase reference value, which is shown by the green dotted line in Figure 4b. However, as demonstrated in Figure 4a,b, the realized MS I transmission is suboptimal. Notably, at the resonant frequency, i.e., 4.3 GHz in Figure 1b, MS I exhibit a transmission error of 0.95 dB and a phase error of 22° relative to the ideal air reference (dotted lines). This non-ideal transmission attenuates and phase-shifts incident waves in the lower frequency range, thereby affecting the absorption performance near the first resonant peak of the double-layer absorber. Similarly, Figure 4c,d show that the reflection performance of MS II in 6–16 GHz band deviates from that of an ideal perfect electric conductor (PEC) ground plane, which requires reflectivity = 0 dB with a 180° phase shift. At its resonant frequencies in Figure 1b, 8.8 GHz and 12.8 GHz, MS II exhibits both reflection errors of 3.9 dB and 4.5 dB, and phase errors of 8° and 40°. Therefore, we can find that the absorption performance changes more significantly near the second and third resonant peaks. These individual non-ideal behaviors in MS I and MS II, compounded by inherent electromagnetic coupling within the integrated unit cell structure, collectively explain the observed performance variations in the dual-layer response in Figure 1c. Nevertheless, despite these deviations, the fundamental absorption mechanisms and principles of the dual-layer structure remain effectively operational across the design bandwidth.

**Figure 5:**
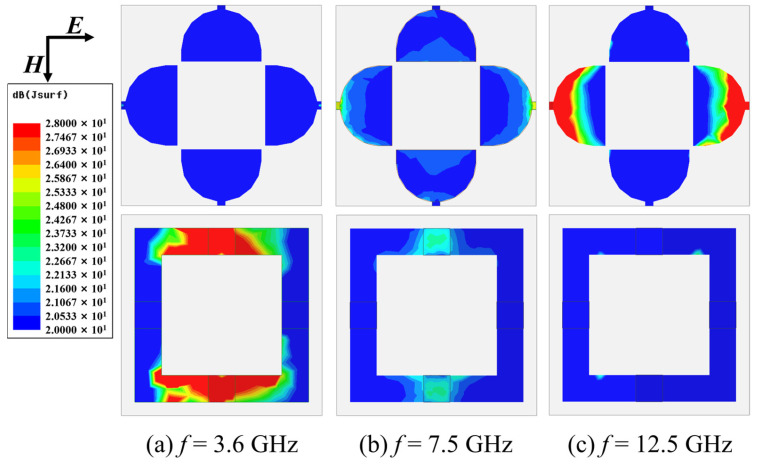
Distribution of induced surface current on the metasurface cells for TE polarization mode at (**a**) 3.6 GHz; (**b**) 7.5 GHz, and (**c**) 12.5 GHz.

To elucidate the electromagnetic energy loss mechanisms of the dual-layer absorber, Figure 5 presents the simulated the distribution of induced surface current on the metasurface cells at three characteristic resonant frequencies, which correspond to Figure 1c. In TE polarization mode, the incident electromagnetic wave electric field direction is horizontal and the induced surface current generated in the absorber is also horizontal, mainly concentrated near the resistive devices. The darker the color, the greater the induced surface current, and the more energy loss is generated. At 3.6 GHz, electromagnetic energy dissipation is predominantly localized within layer II with peak loss concentration at the lumped resistors. For the 7.5 GHz resonance, significant surface currents emerge on both layers, indicating cooperative energy dissipation through coupled ohmic losses. It should also be noted that the color of the induced surface current around the resistors of layer II is darker, indicating that layer II plays a major role in energy loss. At 12.5 GHz, current density concentrates primarily near the tunable resistors of layer I, consistent with prior simulation-based observations. This analysis fundamentally clarifies the origin of the three resonant absorption peaks by directly mapping energy dissipation pathways to specific structural components.

**Figure 6:**
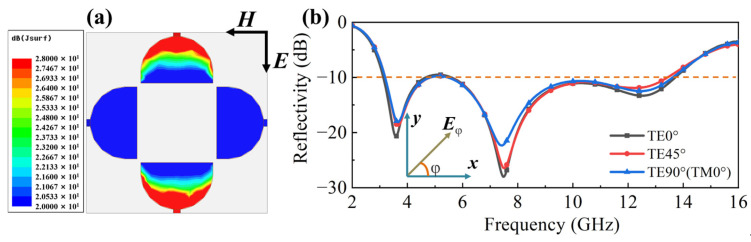
(**a**) Distribution of induced surface current on the layer I for TE90° (TM0°) polarization mode at 12.5 GHz; (**b**) comparison of reflectivity curves with different polarization angles.

**Figure 7:**
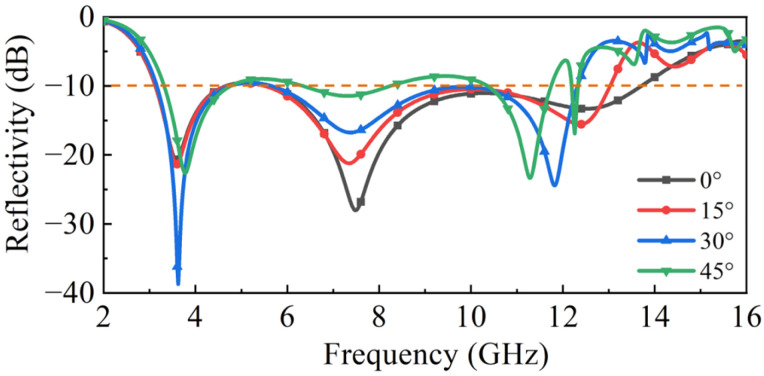
Simulated reflectivity with different oblique incident angles for TE mode.

Furthermore, the polarization independence of the proposed structure was investigated. Figure 6a depicts the simulated distribution of induced surface current on the layer I pattern under TM-polarized incidence at 12.5 GHz. In the TM mode, the electric field direction of the incident electromagnetic wave is along the vertical direction and the induced surface current generated is mainly concentrated near the two resistors in the vertical direction. This distribution aligns closely with the resonant mode observed in Figure 5c, confirming the excitation mechanism. Figure 6b presents the comparison of reflectivity curves with different polarization angles to analyze the polarization independence for the double layer absorber. Here, φ represents the polarization angle. Critically, the three curves exhibit substantial overlap across the operating band. This near-identical response in both induced surface current and spectral characteristics demonstrates that the structure exhibits excellent polarization-independent absorption performance. Such polarization-insensitive performance is a crucial advantage for the practical application of electromagnetic absorbers under different polarized waves, such as linearly polarized and circularly polarized waves, etc. [46]. We discuss next the absorption performance under oblique incidence. We chose the results in Figure 1c as the typical performance to analyze the variational trend in performance with an oblique incident angle. With the oblique incident angle ranging from 0° to 45° for TE polarization mode in Figure 7, the absorption bandwidth of 3.1–12 GHz can be maintained and the amplitude of the reflectivity will slightly weaken but still remain below −10 dB. Note that other peaks appear in the reflectivity above 12 GHz; these are produced by the grating lobe.

### 2.3. Design of Bias Line

**Figure 8:**
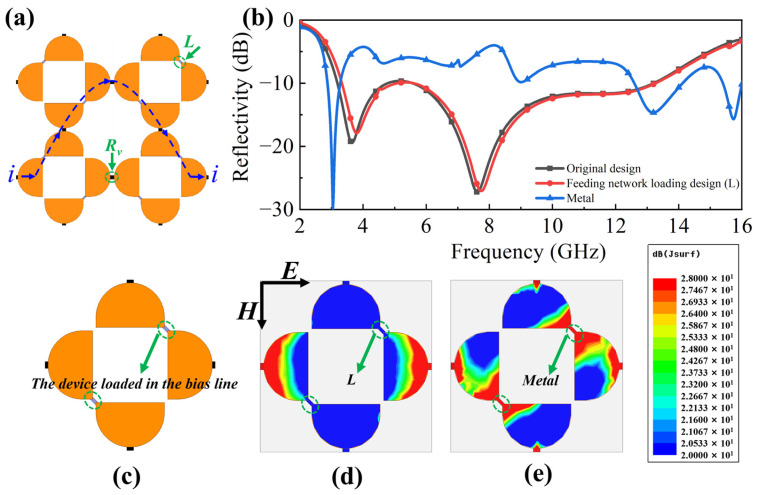
(**a**) Design path of the bias line for the feeding network. (**b**) Comparison of simulated reflectivity between original design and feeding network loading design. (**c**) The loading position of the device on the bias line. Distribution of induced surface current on layer I with (**d**) inductance and (**e**) metal on the bias line.

To fabricate the designed absorber, PIN diodes were employed as substitutes for variable resistors *R_v_*. Consequently, a bias line is required to supply the necessary bias voltages to these diodes. The critical aspect of this bias line design involves connecting the metal patterns across the gap. As illustrated in Figure 8c, the circled area represents the device integrated into the bias line. The specific configuration for the absorber’s bias line is depicted in Figure 8a. The path of the direct current (DC) for the control signal is designed as a parabolic series and the inductors are alternately connected into MS I cells at right or left oblique sites. Within each unit cell, a 110 nH inductor was soldered across the gap between two adjacent cells, as indicated by the arrow in Figure 8a. Utilizing these inductors as high-frequency chokes provides high impedance at the operational high frequency while simultaneously establishing a DC path for the PIN diode control signals. Figure 8b compares the simulated reflectivity between the original design (without bias circuitry) and the feeding network loading design with inductors. It is observed that the reflectivity of the inductor-loaded design can maintain the original absorption performance. If the metal line connecting the units is used to form a bias line, the reflectivity will change completely from the original design without the line. Furthermore, surface current analysis was conducted on the tunable transmission layer with bias lines. Theoretically, placing a metal directly at the circled position in Figure 8c would also permit DC current flow. However, as shown in Figure 8e, a strong current concentration is observed at that location at the operating frequency of 12.5 GHz. Clearly, compared with the distribution in Figure 8d, the current result in Figure 8e is undesirable. In contrast, a comparison between Figure 8d and Figure 5c reveals that the incorporation of inductors does not adversely affect the performance.

### 2.4. Measurement Results

**Figure 9:**
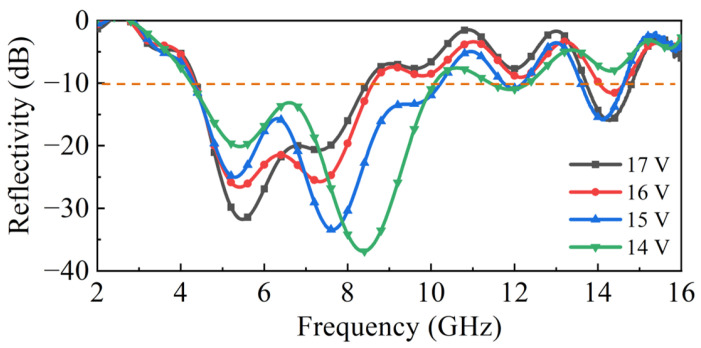
Measured reflectivity curves of the metasurface absorber under a set of DC voltage values.

**Figure 11:**
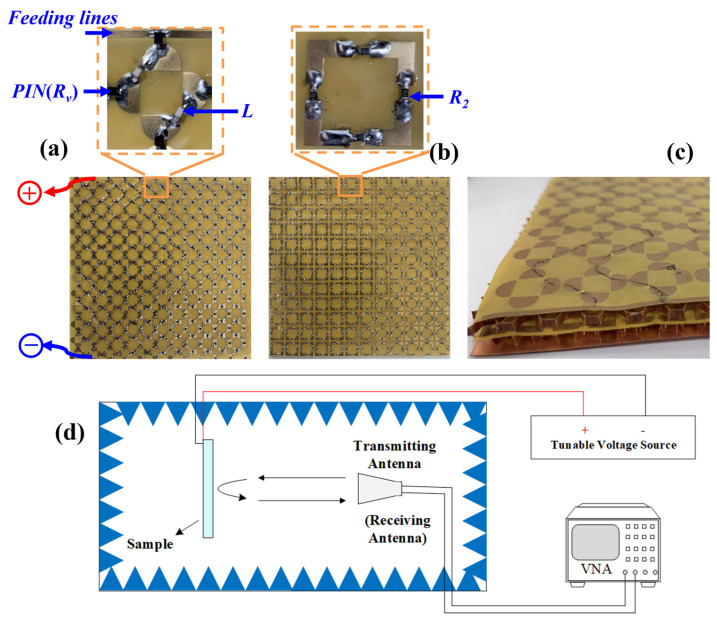
Photograph of the fabricated prototype: (**a**) MS I with the enlarged unit; (**b**) MS II with the enlarged unit; (**c**) a 3D view of the arrangement in layers. (**d**) Schematic diagram of the measurement setup.

In the experimental phase of this study, a prototype of the proposed double-layer structure was fabricated as shown in Figure 11. To implement the variable resistance functionality explored in simulations, the simulated variable resistor was replaced with a practical PIN diode (NXP Semiconductors). The PIN diode exhibits a negative correlation between its resistance and the applied forward bias voltage. Figure 9 presents the measured absorption performance under a range of applied direct current (DC) bias voltages.

As the DC bias voltage was decreased from 17 V to 14 V, the equivalent resistance of the PIN diode correspondingly increased and the reconfigurable metasurface absorber exhibits wideband absorption performance, which can be adjusted continuously below −10 dB, ranging from 4.2 to 14.6 GHz. This increase in resistance induced a distinct shift in the absorption characteristics: the absorption frequency band and amplitude can achieve dynamic reconfigurable effects and the second absorption peak near 8 GHz has the most obvious adjustment effect. Overall, the experimentally observed voltage-dependent tuning behavior aligns well with the resistance-dependent trends predicted by the simulations, as shown earlier in Figure 3b. Both the measured and simulated results have a regulating effect near the second resonant peak of 8GHz. The first resonant peak of the measured result is slightly towards higher frequencies compared to the simulated result, but the position of the first resonant peak of both the measured and simulated results can remain unchanged. Slight differences in the position of the absorption peaks exist between measurements and simulations that result from the variation in junction capacitance and package inductance of the PIN diodes.

## 3. Experiment

### 3.1. Geometric Structure of the Absorber

The functional diagram of the double-layer electromagnetic absorber is illustrated in Figure 1a. The main idea of the absorber is to design each layer into two different functions, and achieve triple-band absorption through the combination of two layers. In the lower frequency band (near 4 GHz), the layer I works as a band-pass filter and transmits electromagnetic waves, and then the layer II combined with the metal plane constitutes a lower frequency absorber. The absorption performance of the second frequency band (near 8 GHz) is the combined effect of layer I and layer II. In the higher frequency band (near 12 GHz), the layer II has strong reflection performance, so it acts as a metal plane, and the layer I combined with the layer II constitutes a higher frequency absorber. Through bonding application of double-layer structure, a combination of the absorption performance at three frequencies is achieved.

**Figure 10:**
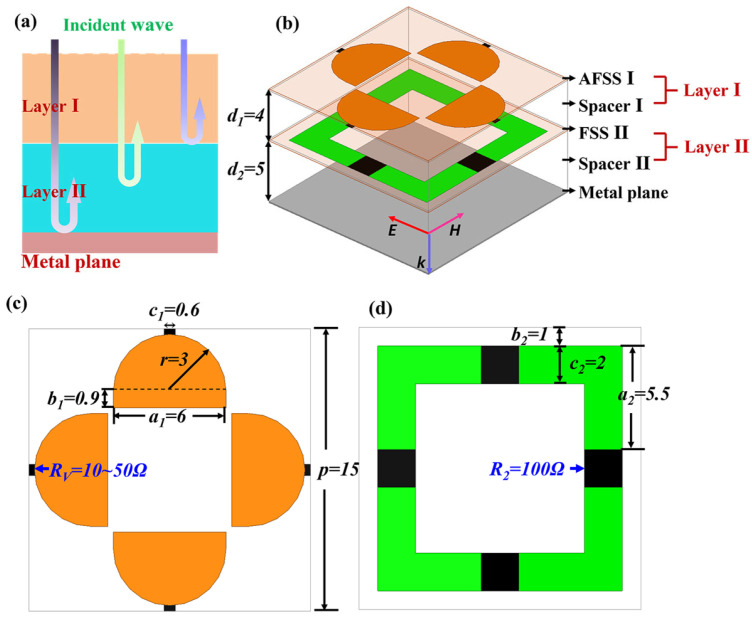
(**a**) Functional diagram of the metasurface absorber; (**b**) schematic 3D view of the metasurface absorber; sizes of the cells of (**c**) MS I and (**d**) MS II (unit: mm).

Figure 10b presents the schematic 3D view of the metasurface absorber, which is made up of two metasurface layers (MS I, MS II), two dielectric spacer layers (spacer I, spacer II), and a metal plane. The FSS I is at the front and the FSS II in the middle separated by the spacer I. The metal plane becomes substrate, which is isolated from the MS II with the spacer II. The layer I in Figure 10a represents the combination of the MS I and the spacer I and the layer II is composed by MS II and spacer II. The MSs are periodic arrays that consist of cells printed on a thin FR4 material with a thickness of 0.15 mm. The dielectric spacers are made from lightweight Nomex^®^ honeycomb. By optimizing the design, Table 1 lists the size parameters of the metasurface absorber cells, which are specifically illustrated in Figure 10c,d in detail. The thickness of the spacers will affect the position and amplitude of the absorption peak. Variable resistor *R_v_*, which is achieved by regulating the PIN diode in sample preparation and lumped resistor *R*_2_ = 100 ohm indicate resistors in the full-wave simulation. To achieve polarization independence, the cells of the metasurfaces are designed as a symmetrical structure and there are four variable resistors (PIN diodes) or lumped resistors for each elementary cell.

### 3.2. Experimental Setup

Based on the above design, a 180 mm × 180 mm reconfigurable metasurface absorber was fabricated. Figure 11a,b show the sample diagrams of MS I and MS II, respectively, as well as the various components in their enlarged units and the feeding lines. Figure 11c shows a 3D view of the sample. The two MS layers were fabricated using standard printed-circuit-board techniques and the resistors are connected inside the cells. The PIN diodes (NXP Semiconductors) function as variable resistors whose values are controlled by a DC signal. The PIN diodes present low resistance at a large forward bias voltage and high resistance at a small voltage.

The reflectivity of the absorber is measured using free-space measurement in the anechoic chamber, as illustrated in Figure 11d. In the free-space measurement process, the broadband double-ridged horn antenna, connected with a vector network analyzer (VNA), is used to measure the reflectivity from the fabricated absorber. In this paper, reflectivity is expressed in dB values. The lower the reflectivity, the better the absorption performance of the metasurface absorber. The reflectivity of −10 dB is equivalent to an absorption rate of 90% and the reflectivity of −20 dB is equivalent to an absorption rate of 99%. The electromagnetic background field of the test site will have a certain impact on the measurement performance. Therefore, the test environment is generally calibrated before the test to eliminate the influence of interference signals on performance.

## 4. Conclusions

This work demonstrates the design, principles, and experimental validation of a dual-layer reconfigurable absorber based on integrated metasurface technology. By synergistically combining an upper active metasurface layer (MS I) incorporating tunable resistors with a lower passive metasurface layer (MS II) loaded with lumped resistors, the absorber achieves effective triple-band absorption at 3.6 GHz, 7.5 GHz, and 12.5 GHz. Crucially, the design enables continuous tuning of the middle resonant frequency from 6.6 GHz to 9 GHz. Simulation results demonstrate that the proposed absorber achieves an exceptionally wide −10 dB reflection bandwidth (indicating >90% absorption) spanning 3.1 GHz to 14.2 GHz. Significantly, in the 6.6–9 GHz frequency band, the reflectivity can be reduced to below −20 dB (exceeding 99% absorption), the intensity of which can be dynamically modulated via the integrated diodes. The excellent agreement between simulation and experimental results confirms the efficacy of our design strategy. This design successfully addresses the bandwidth limitations of single-layer absorbers and integrates reconfigurability within a multilayer structure. We also need to note that the regulation range of tunable absorbers is affected by the parameters of active microwave components. In the future, the lattice control of a single metasurface unit is expected to further enhance the performance of the structure.

## Figures and Tables

**Table 1:** Size parameters of the metasurface absorber cells.

Parameters	*d*_1_	*d*_2_	*p*	*a*_1_	*b*_1_	*c*_1_	*r*	*a*_2_	*b*_2_	*c*_2_
Unit (mm)	4	5	15	6	0.9	0.6	3	5.5	1	2

## Data Availability

The original contributions presented in this study are included in the article. Further inquiries can be directed to the corresponding authors.

## References

[1] Li M., Zhang W. (2023). Design of a Multilayer Wideband Absorber Based on Frequency Selective Surface. Opt. Quant. Electron..

[2] Pan Y., Dong J., Wang M., Luo H., Abdulkarim Y.I. (2023). Inverse Design of Ultra-Wideband Transparent Frequency Selective Surface Absorbers Based on Evolutionary Deep Learning. J. Phys. D Appl. Phys..

[3] Ranjan S.K., Sahoo S. (2024). A Review of Metamaterial-Based Microwave Absorbers and Sensors. J. Electron. Mater..

[4] Celenk E., Lynch C., Tentzeris M.M. (2024). An Ultrawideband All-Textile Metamaterial Absorber for Ku-, K-, and Ka-Band Applications. IEEE Antennas Wirel. Propag. Lett..

[5] Hou Z., Cheng J., Kong X., Zou Y., Pan H., Pang X., Zhao S. (2025). Design of Ultrabroadband Metamaterial Absorber With Angle-Insensitive Characteristics Covering the L, S, C, X, and Ku Bands. IEEE Antennas Wirel. Propag. Lett..

[6] Yang R., Luo Z., Liang J.C., Dai J.Y., Cheng Q., Cui T.J. (2025). Reconfigurable Metasurface with Multiple Functionalities of Frequency-Selective Rasorber, Frequency-Selective Surface, Absorber, and Reflector. Adv. Mater. Technol..

[7] Almawgani A.H.M., Srilatha K., Madhav B.T.P., Venkatesh B., Sravan C.V.S.A., Rao M.C., Alhawari A.R.H. (2023). Dual Band Metasurface Absorber with Insensitive Polarization and Incidence Angle for S and C Band Applications. J. Commun. Technol. Electron..

[8] Liu X., Zhang Y., Li M., Feng C., Zhao Y. (2023). Polarization and Angular Insensitive Perfect Metasurface Absorber in Near-Ultraviolet Region. J. Nanophotonics.

[9] Weng Z., Guo Y. (2019). Broadband Perfect Optical Absorption by Coupled Semiconductor Resonator-Based All-Dielectric Metasurface. Materials.

[10] Wu R.Y., He S., Wu J.W., Bao L., Cui T.J. (2023). Multi-Frequency Amplitude-Programmable Metasurface for Multi-Channel Electromagnetic Controls. Nanophotonics.

[11] Guo X., Luo Y., Chen Z.N., Yan N., An W., Ma K. (2023). Multiple Tri-Beam Low Scanning Loss Metasurface Antenna for Wide-Angle Coverage by Superposing Focusing and Periodic Phases. IEEE Trans. Microw. Theory Techn..

[12] Wang J., Zhao X.-C., Jiang Y.-N., Gu W.-Q., Xu K.-D. (2024). Optimal Design of Broadband Linear-to-Circular Polarization Conversion Metasurface. Mater. Des..

[13] Yuan X., Zhou H., Ye X., Zhang R., Chen M., Zhang X., Li W., Chen X., Li L., Huang Y. (2022). Impact of Power Spectrum in Geometrical Coding on the Scattering of Random Electromagnetic Coding Metasurface. IEEE Trans. Antenn. Propag..

[14] Liu X., Shu M., Chen X., Zhang A. (2020). Four-Dimensional Characteristic Matrix for Electromagnetic Coupling of Multilayer Sub-Wavelength Metasurface System. IEEE Trans. Electromagn. Compat..

[15] Khan H.A., Majeed A., Zahra H., Kakepoto F.G., Abbas S.M., Alathbah M. (2024). Transparent Conformal Metasurface Absorber for Ultrawideband Radar Cross Section Reduction. J. Phys. D Appl. Phys..

[16] Su J., Li W., Qu M., Yu H., Li Z., Qi K., Yin H. (2022). Ultrawideband RCS Reduction Metasurface Based on Hybrid Mechanism of Absorption and Phase Cancellation. IEEE Trans. Antenn. Propag..

[17] Peng T., Huang W., Zhou X., Zhou T., Xi Q., Yang L. (2024). Ultrathin and Omnidirectional Absorbing Metasurface Designed for Electromagnetic Shielding of Discrete Sources. IEEE Antennas Wirel. Propag. Lett..

[18] An J., Xu C., Ng D.W.K., Alexandropoulos G.C., Huang C., Yuen C., Hanzo L. (2023). Stacked Intelligent Metasurfaces for Efficient Holographic MIMO Communications in 6G. IEEE J. Sel. Areas Commun..

[19] Naveed M.A., Bilal R.M.H., Baqir M.A., Bashir M.M., Ali M.M., Rahim A.A. (2021). Ultrawideband Fractal Metamaterial Absorber Made of Nickel Operating in the UV to IR Spectrum. Opt. Express.

[20] Liu Z., Guo L., Zhang Q. (2020). Design of Dual-Band Terahertz Perfect Metamaterial Absorber Based on Circuit Theory. Molecules.

[21] Li J., Yuan Y., Wu Q., Zhang K. (2024). Bi-Isotropic Huygens’ Metasurface for Polarization-Insensitive Cross-Polarization Conversion and Wavefront Manipulation. IEEE Trans. Antenn. Propag..

[22] Fu C., Zhang L., Liu L., Dong S., Yu W., Han L. (2023). RCS Reduction on Patterned Graphene-Based Transparent Flexible Metasurface Absorber. IEEE Trans. Antenn. Propag..

[23] Luo H., Wei S., Zhang H., Gong K., Liu Q. (2025). A C-Band Polarization Conversion Metasurface Antenna with Broadband RCS-Reduction. IEEE Antennas Wirel. Propag. Lett..

[24] Wu Y., Fu C., Jiang Y., Bian J., Gu W. (2025). Angle-Insensitive Broadband Transparent Microwave Absorber with High Shielding Effectiveness. Opt. Lett..

[25] Patel S.K., Parmar J., Katkar V. (2022). Graphene-Based Multilayer Metasurface Solar Absorber with Parameter Optimization and Behavior Prediction Using Long Short-Term Memory Model. Renew. Energ..

[26] Lei Y., Jia X., Zhang J., Hu J., Kang Y., Zou T. (2024). An Ultra-Broadband Multi-Layer Dielectric Gradient Honeycomb Microwave Absorber Enhanced by the Double Layer Metasurface. Opt. Mater..

[27] Wen J., Ren Q., Peng R., Zhao Q. (2022). Multi-Functional Tunable Ultra-Broadband Water-Based Metasurface Absorber with High Reconfigurability. J. Phys. D Appl. Phys..

[28] Darvishi Bahloli M., Bordbar A., Basiri R., Jam S. (2022). A Tunable Multi-Band Absorber Based on Graphene Metasurface in Terahertz Band. Opt. Quant. Electron..

[29] Bhattarai K., Silva S., Song K., Urbas A., Lee S.J., Ku Z., Zhou J. (2017). Metamaterial Perfect Absorber Analyzed by a Meta-Cavity Model Consisting of Multilayer Metasurfaces. Sci. Rep..

[30] Zhang K.-L., Hou Z.-L., Bi S., Fang H.-M. (2017). Modeling for Multi-Resonant Behavior of Broadband Metamaterial Absorber with Geometrical Substrate. Chin. Phys. B.

[31] Ye Y., Yu S., Li H., Gao Z., Yang L., Zhao T. (2022). Triple Fano Resonances Metasurface and Its Extension for Multi-Channel Ultra-Narrow Band Absorber. Results Phys..

[32] Zhang S., Wen F., Zhai M., Li Z., Ye H., Zhang H., Gu Y., Lei Y., Wang W., Zhang Y. (2024). Terahertz Dynamic Multiband Perfect Absorber with a Digital Coding Graphene-Diamond Metasurface. Phys. Rev. Appl..

[33] Wu Z., Ren Z., Wang J., Hou S., Liu Y., Zhang Q., Mao J., Liu X., Cao F. (2022). Realization of an Efficient Wide-Angle Solar Selective Absorber via the Impedance Matching. Sol. Energ. Mater. Sol. Cell.

[34] Liu Y., Huang R., Ouyang Z. (2021). Terahertz Absorber with Dynamically Switchable Dual-Broadband Based on a Hybrid Metamaterial with Vanadium Dioxide and Graphene. Opt. Express.

[35] Li W., Ma J., Zhang H., Cheng S., Yang W., Yi Z., Yang H., Zhang J., Wu X., Wu P. (2023). Tunable Broadband Absorber Based on a Layered Resonant Structure with a Dirac Semimetal. Phys. Chem. Chem. Phys..

[36] Chung M., Jeong H., Kim Y.-K., Lim S., Baek C.-W. (2022). Design and Fabrication of Millimeter-Wave Frequency-Tunable Metamaterial Absorber Using MEMS Cantilever Actuators. Micromachines.

[37] Wang B.-X., Xu C., Duan G., Xu W., Pi F. (2023). Review of Broadband Metamaterial Absorbers: From Principles, Design Strategies, and Tunable Properties to Functional Applications. Adv. Funct. Mater..

[38] Azad A.K., Taylor A.J., Smirnova E., O’Hara J.F. (2008). Characterization and Analysis of Terahertz Metamaterials Based on Rectangular Split-Ring Resonators. Appl. Phys. Lett..

[39] Mou N., Tang B., Li J., Zhang Y., Dong H., Zhang L. (2021). Demonstration of Thermally Tunable Multi-Band and Ultra-Broadband Metamaterial Absorbers Maintaining High Efficiency during Tuning Process. Materials.

[40] Kowerdziej R., Wróbel J., Kula P. (2019). Ultrafast electrical switching of nanostructured metadevice with dual-frequency liquid crystal. Sci. Rep..

[41] Sbeah Z.A., Adhikari R., Sorathiya V., Chauhan D., Chang S.H., Dwivedi R.P. (2023). A Review on Metamaterial Sensors Based on Active Plasmonic Materials. Plasmonics.

[42] Wang J., Yang R., Ma R., Tian J., Zhang W. (2020). Reconfigurable Multifunctional Metasurface for Broadband Polarization Conversion and Perfect Absorption. IEEE Access.

[43] He S., Yang H., Jiang Y., Deng W., Zhu W. (2019). Recent Advances in MEMS Metasurfaces and Their Applications on Tunable Lens. Micromachines.

[44] Deng R., Zhang K., Li M., Song L., Zhang T. (2019). Targeted Design, Analysis and Experimental Characterization of Flexible Microwave Absorber for Window Application. Mater. Des..

[45] Ghosh S., Srivastava K.V. (2015). An Equivalent Circuit Model of FSS-Based Metamaterial Absorber Using Coupled Line Theory. IEEE Antennas Wirel. Propag. Lett..

[46] Ahmed F., Hassan T., Shoaib N. (2020). A Multiband Bianisotropic FSS with Polarization-Insensitive and Angularly Stable Properties. IEEE Antennas Wirel. Propag. Lett..

